# IMPACT OF FRAILTY ON THE DIGNITY OF COMMUNITY-DWELLING OLDER PEOPLE

**DOI:** 10.1093/geroni/igac059.981

**Published:** 2022-12-20

**Authors:** Ali Darvishpoor Kakhki, Fereshteh Moradoghli, Roghayeh Esmaeili

**Affiliations:** Shahid Beheshti University of Medical Sciences, Tehran, Tehran, Iran; Shahid Beheshti University of Medical Sciences, Tehran, Tehran, Iran; Shahid Beheshti University of Medical Sciences, Tehran, Tehran, Iran

## Abstract

The population of people aged 60 and older is rapidly increasing in developing countries such as Iran due to declining birth rates and increased life expectancy. Old age is associated with increased risk for frailty and reduced dignity. This study investigated the impact of frailty on the dignity of older people in Tehran, Iran. This cross-sectional study was conducted on 200 individuals aged 60 years and older. Data collection relied on the Demographic Questionnaire, Frailty Index for Elders (FIFE) and the Patient Dignity Inventory (PDI). Data were analyzed with SPSS 25. The mean age of the participants was 68 (±5.05) years; 62% of the participants were at risk for frailty, and 69% had few dignity-related problems. The multiple regression results showed that frailty was significantly associated with dignity (ß = -0.571, p < 0.001). The association was significant across all the dimensions of dignity measured by the PDI. The highest predictors of frailty included dependency (ß = -0.584, p < 0.001), followed by existential distress (ß = -0.560, p < 0.001), symptom distress (ß = -0.400, p = 0.400), social support (ß = -0.391, p < 0.001), and peace of mind (ß = -0.338, p < 0.001) in dignity. The results show that higher levels of frailty in older people are associated with decreases in their dignity, and frailty was the leading predictor of dignity. Providers should develop programs to prevent and reduce frailty in those at risk and to enhance the dignity of the already frail.

